# Fibroscanning Recommended by NICE in Drug and Addiction Services: An Audit

**DOI:** 10.1192/bjo.2024.623

**Published:** 2024-08-01

**Authors:** Fiona Atkinson, David Robertson, Ivan Saeger, Michael Kelleher

**Affiliations:** South London and Maudsley NHS Foundation Trust, London, United Kingdom

## Abstract

**Aims:**

The prevalence and subsequent physical health burden of alcohol use disorder is on the increase in almost all age groups in the UK and nearly 1 in 5 of the population will drink at hazardous levels. Those who drink heavily often have limited or patchy engagement with physical health services and improving this should be a focus of drug and alcohol services.

Our aim was to audit the proportion of clients attending the alcohol service at Lorraine Hewitt House (LHH) who had completed a fibroscan, to audit the outcomes of those fibroscans and to audit the outcomes of the onward referrals where they had been made.

**Methods:**

Since starting the liver clinic, more than 100 fibroscans have been completed. These are typically offered to clients in the alcohol pathway and where it can be facilitated, they are done on the day, or otherwise booked in for scheduled appointments.

We audited the results of the scans, for liver stiffness and liver steatosis. Additionally, for those who had abnormal results requiring onward referral, we audited the outcomes of these referrals.

Outcomes and overall physical health were subsequently discussed with each patient who underwent a liver scan.

**Results:**

A total of 100 fibroscans were audited. This represents approximately one third of the clients with alcohol as their primary problem substance at LHH.

Every client had the results of their scan explained and discussed with them and were given lifestyle advice and interventions.

A total of 37 scans (37%) had all their parameters within the normal range. 16 (16%) showed an increased liver stiffness of >10kPa and 15 people (15%) gave consent and were subsequently referred to our local liver clinic. 13% had stage 1 steatosis (238–260dB/M), 18% had stage 2 steatosis (260–290dB/M) and 29% had stage 3 steatosis (>290/dB/M).

Of the 15 referrals made to liver clinic, 10 (66.7%) attended their liver clinic follow up appointments and 9 of these clients are awaiting further interventions – the remaining 1 client has been discharged back to their GP.

Of the remaining 5 referrals made to liver clinic, 2 are awaiting appointment dates and 3 are pending triage.

**Conclusion:**

The physical health of clients attending drug and addiction services is often complicated and in need of specific and targeted interventions. Liver health is particularly relevant to the alcohol client group and integrating fibroscans into drug and addiction services facilitates better engagement and early assessment and intervention.

